# Thermally provoked itch in atopic dermatitis: current evidence, knowledge gaps, and digital approaches to nocturnal scratching

**DOI:** 10.3389/fmed.2026.1935603

**Published:** 2026-10-22

**Authors:** Dengmei Xia, Yue Yuan, Jinghong Huang

**Affiliations:** 1 Department of Dermatology, West China Second University Hospital, Sichuan University, Chengdu, China; 2 Key Laboratory of Birth Defects and Related Diseases of Women and Children, Sichuan University, Ministry of Education, Chengdu, China

**Keywords:** atopic dermatitis, digital health, neuroimmune mechanisms, scratch monitoring, thermally provoked itch

## Abstract

Atopic dermatitis (AD) is a chronic inflammatory skin disease in which pruritus significantly contributes to sleep disturbance, impaired quality of life, and the itch–scratch cycle. Heat, sweating, hot-water exposure, and nocturnal occlusion are frequently reported as itch-aggravating factors, although these exposures are biologically heterogeneous. This narrative review critically examines the current clinical relevance, mechanistic evidence, and objective monitoring of thermally provoked itch in AD. Thermosensitive ion channels, epithelial cytokines, neuropeptides, dermal fibroblast-derived artemin, and altered peripheral and central sensory processing provide a plausible framework for thermally provoked itch. However, the relative contribution of these pathways to heat-aggravated itch in humans remains incompletely defined. Digital monitoring technologies, including wrist-worn actigraphy, smartwatch-based algorithms, hand-mounted sensors, non-wearable systems, thermal imaging, acoustic sensing, and multimodal devices, are increasingly used to quantify scratch duration, frequency, intensity, and sleep-related patterns in real-world settings. These tools do not replace patient-reported outcomes, but they may provide an objective behavioral layer for assessing otherwise hidden nocturnal burden and monitoring changes over time. Future prospective studies should define heat-responsive clinical characteristics, standardize nocturnal scratch endpoints, validate digital measures across pediatric and adult populations, and jointly assess thermal and humidity exposure, skin temperature, itch, scratching, sleep, skin-barrier status, disease severity, and relevant confounders. Such studies are needed to determine whether thermal exposure independently predicts clinically meaningful nocturnal scratching and to establish the clinical value of digital scratch-monitoring endpoints in AD. Integrating mechanistic insights with objective monitoring may support more precise assessment and patient-centered management of AD.

## 1 Introduction

Atopic dermatitis (AD) is a chronic relapsing inflammatory skin disease characterized by intense pruritus. Itch repeatedly interrupts sleep, concentration, and daily routines. The sensation of itch in AD initiates the itch–scratch cycle, which exacerbates skin barrier impairment and inflammation, thereby worsening the overall condition. A systematic review including approximately 189,000 patients from 13 studies found that poor sleep quality in AD is closely associated with higher disease severity and greater pruritus intensity, with itch emerging as the strongest predictor of sleep disturbances (1). Nighttime pruritus can also disrupt sleep patterns, circadian rhythms, and emotional regulation, creating a loop in which inflammation, scratching, and psychological distress feed into one another (2, 3). Therefore, effective itch control is central to AD management.

Environmental conditions may influence symptom burden in AD, and rising ambient temperatures provide a broader context for examining heat-aggravated itch (4). However, this review focuses on thermo-sensation, itch, nocturnal scratching, and digital monitoring rather than on the epidemiology of climate change and AD outcomes. Patients commonly report worsening itch in warm environments or after hot-water exposure, and experimental studies indicate that skin heating can modify itch perception (4, 5). Nevertheless, direct evidence linking ambient temperature or heat events to objectively measured nocturnal scratching, AD flare occurrence, or healthcare utilization remains limited. The mechanisms through which mild warmth provokes or aggravates itch in susceptible patients therefore remain incompletely understood.

Pruritus is commonly assessed using patient-reported outcomes, such as the visual analog scale (VAS) or numeric rating scales, which may be affected by recall bias and are especially limited for nocturnal symptoms and young children (6). Meanwhile, AD has a high incidence in infancy and early childhood, a population in which descriptions of pruritus are often imprecise and unreliable. These assessments also overlook much of what occurs during sleep, when patients may scratch without awareness. Digital health technologies can continuously record scratching in the home environment and may provide an objective behavioral measure that complements patient-reported outcomes (7–9).

Although AD has been extensively reviewed from immunological and neurobiological perspectives, thermally provoked itch has received less focused attention. Recent advances in digital scratch monitoring have primarily been discussed in terms of device design, sensor performance, and algorithmic accuracy, with less emphasis on how objective scratching metrics may be interpreted within trigger-specific clinical contexts. This review aims to bridge these areas by integrating heat-sensitive itch mechanisms, nocturnal scratching behavior, and emerging digital endpoints into a translational framework for AD care.

## 2 Literature search and scope of this narrative review

This article is a narrative, critical review rather than a systematic review or meta-analysis. Relevant literature was identified through searches of PubMed, Web of Science, and Google Scholar from database inception to 28 February 2026. Search terms included atopic dermatitis, itch or pruritus, heat, warmth, sweating, thermo-sensation, nocturnal scratching, actigraphy, wearable sensors, smartwatch-based monitoring, thermal imaging, acoustic sensing, digital biomarkers, and digital health. We considered peer-reviewed English-language original studies, experimental human studies, mechanistic and preclinical studies, observational clinical studies, device development and validation studies, clinical trials, and relevant reviews. References were selected according to their relevance to thermally provoked itch, neuroimmune mechanisms, nocturnal scratching and sleep disturbance, or objective digital approaches to scratch monitoring in AD. No formal risk-of-bias assessment or quantitative synthesis was undertaken.

## 3 Clinical association and characteristics of atopic dermatitis and thermally provoked itch

### 3.1 Epidemiology and clinical manifestations of heat stimuli as itch triggers

For this review, thermally provoked itch refers to itch perceived to be triggered or aggravated by increased ambient temperature, increased skin temperature, or heat-retaining environmental conditions. This working definition is intended to organize a heterogeneous literature and does not imply that temperature is the sole causal factor. In clinical reports, hot-water exposure, physical exertion, sweating, and bedding-related nocturnal occlusion are often grouped with warmth, although these exposures are not biologically or clinically interchangeable (10–12). Sweating may alter local humidity, sweat composition, and evaporative cooling; hot-water exposure can affect skin temperature, hydration, surface lipids, and barrier function; and nocturnal bedding may increase both heat retention and local humidity (13, 14). Accordingly, associations between these thermal-environmental contexts and itch should not be interpreted as evidence that temperature alone is responsible.

Clinically, the itch induced by heat in individuals with AD is often described as a sharp, stinging sensation that qualitatively differs from the normal baseline itch (15). This clinical variability is important: Heat sensitivity likely reflects a mixture of barrier status, inflammatory activity, neural sensitization, and individual predisposition. Observational studies have reported associations between sensitivity to thermally provoked itch and disease activity, with heightened sensitivity observed during acute flare-ups of AD (5, 16). This makes thermally provoked itch a possible clinical correlate of active disease, but it should not be regarded as a uniform symptom across all patients.

Most clinical evidence relies on patient-reported triggers and observational associations, and few studies have simultaneously quantified thermal exposure, itch intensity, and subsequent scratching behavior in the same individuals. Consequently, the independent contribution of temperature—compared with humidity, sweating, occlusion, and barrier-related factors—remains uncertain. These limitations also prevent the classification of heat-responsive clinical characteristics. Reported heat sensitivity may indicate a heat-responsive clinical characteristic, a heat-aggravated itch pattern associated with active disease, or greater overall disease severity rather than a biologically distinct subgroup. Establishing a clinically meaningful characteristic would require reproducibility across cohorts, temporal stability, discriminatory validity beyond overall disease activity, and preferably differences in prognosis or treatment response. Until such evidence is available, more cautious terminology is warranted.

### 3.2 Amplification role of thermally provoked itch in the itch–scratch cycle

Thermal stimuli not only directly stimulate pruriceptive neural pathways but also trigger a series of events that exacerbate the itch–scratch cycle in AD. The mechanical damage caused by scratching, which is triggered by thermally provoked itch compromises the skin barrier, resulting in the release of endogenous alarm signals, including interleukin-33 (IL-33) and thymic stromal lymphopoietin (TSLP) (17). These cytokines serve as powerful pro-inflammatory agents that attract and activate immune cells, heightening local inflammation. Simultaneously, neuropeptides such as substance P (SP) and calcitonin gene-related peptide (CGRP) are released from sensory nerve endings, further enhancing neurogenic inflammation and vasodilation (18, 19). This inflammatory environment lowers the activation threshold of sensory neurons, making them hypersensitive to subsequent thermal and pruritic stimuli (20). As a result, a detrimental positive feedback loop arises, characterized by a cycle of “heat–itch–inflammation–more itch,” which perpetuates and worsens disease pathology. Importantly, the nocturnal setting plays a significant role in this cycle; the increase in microenvironmental temperature due to bedding and covers during sleep can exacerbate thermally provoked itch and scratching behaviors at night (13). This nighttime aggravation is frequently overlooked yet significantly contributes to sleep disturbances and reduced quality of life in patients with AD. The interaction between thermal stimuli, neuroimmune activation, and behavioral responses highlights the intricate nature of itch pathophysiology in AD and emphasizes the necessity for comprehensive therapeutic strategies that address both environmental and molecular factors contributing to thermally provoked itch.

Although the heat–itch–scratch–inflammation loop is biologically plausible, direct longitudinal evidence linking heat-triggered scratching to barrier deterioration, inflammatory escalation, or subsequent AD flare remains limited. Future studies should therefore test whether thermal triggers can predict clinically meaningful changes in disease trajectory rather than serving only as subjective aggravating factors.

## 4 Pathophysiological mechanisms of thermally provoked itch in AD

Evidence relevant to thermally provoked itch in AD comes from study designs that address distinct questions and should not be interpreted as equivalent. In this review, preclinical and animal studies are considered hypothesis-generating for biological mechanisms (Figure 1), whereas controlled human experimental studies provide evidence of sensory or physiological responses under defined conditions. Observational patient studies can identify clinical associations but cannot establish causality. Prospective longitudinal and interventional studies are needed to evaluate temporal relationships, therapeutic relevance, and clinical significance. Digital-device studies are considered separately, as analytical validation against an appropriate reference standard establishes detection performance but does not demonstrate clinical validity, responsiveness to treatment, or relevance to thermal exposure.

**Figure 1:**
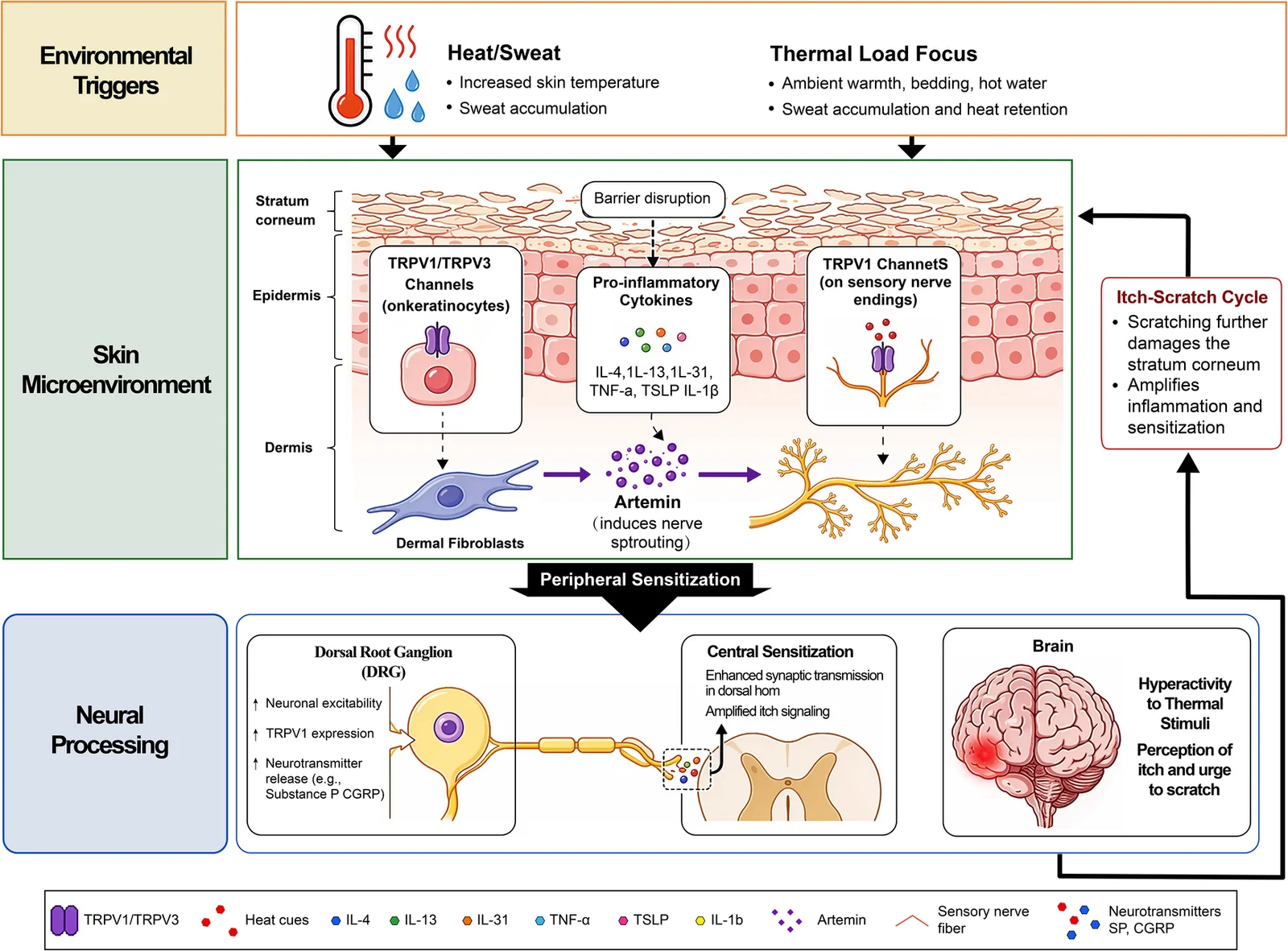
Proposed neuroimmune and cellular pathways potentially relevant to thermally provoked itch in atopic dermatitis.

### 4.1 Neurobiological pathways and transient receptor potential channel activation

Thermally provoked itch in AD arises at the interface between keratinocytes, sensory neurons, and inflammatory mediators (Table 1). The transient receptor potential vanilloid 1 (TRPV1) channels detect noxious heat and chemical irritants and are expressed in peripheral sensory neurons, keratinocytes, and fibroblasts (21–23). Current evidence suggests that increased skin temperature and noxious heat exposure can modulate itch through thermosensitive pathways, although the role of TRPV1 in mild warmth-provoked itch in human AD remains incompletely defined (24). However, TRPV1 alone is unlikely to explain the full spectrum of heat-sensitive itch, particularly when mild warmth is sufficient to provoke symptoms. Calcitonin gene-related peptide (CGRP)-expressing sensory neurons can encode both heat and itch, whereas vesicular glutamate transporter 2 (VGLUT2)-mediated input from TRPV1 afferents has been proposed to suppress spinal itch transmission through GRPR-related circuitry (25). Whether impairment of this inhibitory pathway contributes to thermally provoked itch in human AD has not been established. Human lesional skin and AD-like animal model studies suggest that enhanced TRPV3 signaling may promote warmth-responsive keratinocyte-derived pruritogenic signaling (26–28). If this inhibitory gate weakens in AD, heat may lose its counter-irritant effect and instead become an itch trigger. TRPV3 is a thermosensitive Ca^2^⁺-permeable channel that is highly expressed in subunit interfaces controlling gating equilibrium and thermal sensitization (26). However, evidence from AD-like mouse models should be interpreted as mechanistic support rather than as evidence of therapeutic efficacy for warmth-aggravated pruritus in human AD.

**Table 1:** Proposed neuroimmune and cellular mechanisms potentially relevant to thermally provoked itch in AD.

Mechanism	Key cells or mediators	Putative role in thermally provoked itch	Evidence specific to thermal provocation in AD	Predominant evidence types and level	Clinical or translational implication
TRPV1-mediated heat sensing	Sensory neurons, keratinocytes, fibroblasts; TRPV1, neuropeptides	Detects noxious heat and chemical stimuli and may amplify neurogenic inflammation in inflamed skin	Indirect or limited; direct causal evidence in human AD is lacking (21–25)	Preclinical and experimental evidence	Biologically plausible contributor to heat-sensitive itch; therapeutic relevance in human AD remains unproven
TRPV3 activation in keratinocytes	Keratinocytes; TRPV3, Ca2 + influx, TSLP, NGF, PGE2	May link innocuous warmth to keratinocyte-derived pruritogenic signaling	Human lesional skin evidence and AD-like animal model evidence; clinical interventional evidence is lacking (26–28)	Human tissue studies and preclinical AD-like models	Supports a plausible warmth-responsive epithelial mechanism; therapeutic relevance in human AD remains unproven
Fibroblast-derived artemin signaling	Dermal fibroblasts, sensory nerves; artemin, substance P	May promote sensory nerve sensitization and lower the warmth threshold for warmth-provoked itch	Human lesional skin observations and mechanistic evidence; longitudinal clinical validation is limited (31, 32)	Human tissue observations and preclinical mechanistic evidence	Suggests a possible stromal- neural contribution; clinical relevance requires further study
Epithelial cytokine-driven sensitization	Keratinocytes, type 2 immune cells; IL-33, TSLP, IL-4, IL-13	Barrier disruption and epithelial cytokines may intensify local inflammation and sensitize itch pathways	Indirect; evidence is largely related to general AD inflammation and itch rather than thermal provocation specifically (17, 20)	General AD immunology and mechanistic studies	Provides contextual AD biology; a heat-specific link to nocturnal scratching or subsequent flare has not been established
Neuropeptide-mediated neurogenic inflammation	Sensory nerve endings, mast cells, endothelial cells; substance P, CGRP	Thermal stimulation and scratching may promote neuropeptide release, vasodilation, and immune-neural amplification	Indirect; the thermal specificity of these pathways in human AD is incompletely established (18, 19)	Neuroimmune itch studies and AD pathophysiology evidence	May help explain burning, stinging, or sharp itch sensations reported by patients with heat-aggravated AD. Direct clinical evidence for thermally provoked itch is limited
Impaired counter-irritation and central sensitization	Spinal cord circuits, brain itch-processing networks; GRPR-related pathways, VGLUT2-associated signaling	Noxious heat or scratching may inhibit itch in AD, allowing heat to aggravate symptoms	Experimental evidence in AD supports altered itch inhibition; however, the association with nocturnal scratching remains untested (25, 29)	Experimental human psychophysical and neurophysiological studies	Supports altered sensory modulation, but there is no confirmed pathway from heat exposure to nocturnal scratching
Sweating, occlusion, and the nocturnal microenvironment	Sweat glands, skin barrier, bedding-related heat retention, and local humidity	Sweating, increased skin temperature, and occlusion may co-occur and intensify itch	Patient-reported and observational evidence indicate that the independent effect of temperature cannot be reliably separated from humidity, sweat, or barrier effects (13, 14)	Observational patient studies and nocturnal itch literature	Supports environmental and behavioral interventions, including temperature control and optimization of the sleep environment
Itch–scratch–barrier feedback loop	Keratinocytes, immune cells, and sensory nerves, alarmins, cytokines, and neuropeptides	Scratching may damage the barrier and further amplify inflammation and itch	Indirect thermal provocation of itch; direct longitudinal evidence linking heat-triggered scratching to subsequent flare is limited (17–20)	Integrated AD pathophysiology, along with experimental and observational evidence	Provides contextual AD biology; however, a heat-specific link to nocturnal scratching or subsequent flare has not been established

AD, atopic dermatitis; CGRP, calcitonin gene-related peptide; GRPR, gastrin-releasing peptide receptor; IL, interleukin; NGF, nerve growth factor; PGE2, prostaglandin E2; TSLP, thymic stromal lymphopoietin; TRPV, transient receptor potential vanilloid; VGLUT2, vesicular glutamate transporter 2. The table provides a critical narrative overview. Evidence from preclinical models, experimental human studies, observational patient studies, and clinical intervention studies should not be interpreted as equivalent. Direct evidence linking quantified thermal exposure to objectively measured nocturnal scratching in AD remains limited.

Neurophysiological studies suggest that thermally provoked itch in AD is not solely cutaneous. Patients with AD show distinct brain hyperactivity to electrical stimuli and delayed thermal-evoked potentials compared with healthy individuals (29). These findings suggest small-fiber dysfunction or altered central processing, rather than just a cutaneous phenomenon (30). In AD, the threshold for itch is often reduced, so mild warmth—which may be perceived as pleasant on healthy skin—can trigger a strong urge to scratch. These observations indicate that the therapeutic implication is not simply to block a single ion channel, but to understand how peripheral sensitization and central modulation jointly lower the threshold for heat-evoked itch.

Nevertheless, thermally provoked itch is unlikely to be explained by a single ion channel or sensory pathway. The relative contributions of TRPV1, TRPV3, keratinocyte-derived mediators, and spinal inhibitory circuits may vary according to disease stage, lesion status, and individual sensory phenotype.

### 4.2 The role of artemin and dermal fibroblasts in thermal hyperalgesia

Beyond direct thermosensitive channel activation, the structural and biochemical environment of the dermis may contribute to thermal hypersensitivity. Dermal fibroblasts from patients with AD have been reported to secrete artemin in response to inflammatory mediators such as substance P (31). Artemin can promote nerve sprouting and thermal hyperalgesia in experimental systems, and its accumulation in lesional AD skin may contribute to altered warmth sensitivity (31, 32). However, its contribution to routine clinical heat-aggravated itch, nocturnal scratching, or treatment response remains uncertain.

This mechanism is consistent with clinical observations that heat and sweating are common itch-aggravating factors in AD, although the relationship is unlikely to be linear for all patients (14). Some patients notice dryness and dermatitis before itch worsens, whereas others experience itch before visible skin inflammation becomes prominent. Circadian or evening changes in autonomic activity may also interact with artemin-related thermal sensitivity, although this connection remains insufficiently defined. New therapeutic approaches for atopic itch increasingly focus on signaling pathways in the skin and dorsal root ganglia (33). In this context, fibroblast-neuron signaling provides a plausible route through which inflamed skin may become unusually responsive to warmth.

Artemin therefore offers a compelling link between dermal inflammation and altered thermal sensation, but its role in routine clinical thermally provoked itch remains incompletely defined. Further studies are needed to determine whether artemin expression identifies a specific heat-sensitive subgroup of patients with AD or primarily reflects broader lesional inflammation.

### 4.3 Peripheral and central sensitization to heat stimuli and histamine-induced pruritus

Sensitization is a key feature distinguishing AD-associated itch from normal itch physiology. In healthy volunteers, noxious heat and repeated scratching can inhibit itch through counter-irritation (34). However, in patients with AD, this inhibitory mechanism appears to be impaired. Research has shown that repetitive scratching and noxious heat fail to inhibit histamine-induced itch in patients with AD, suggesting both peripheral and central sensitization (35). This lack of inhibition may help explain why scratching, although temporarily relieving itch, can contribute to subsequent itch amplification.

Questionnaire-based studies provide clinical support for this mechanism. More than half of patients with AD report heat sensation as part of their itch experience (36). Itch is also frequently worse at night and is aggravated by sweating, physical exertion, and stress, all of which can raise body temperature or alter heat dissipation. The affective burden is clinically important, as peak itch intensity can be deeply distressing and may be perceived as more bothersome than other common pruritic stimuli, such as mosquito bites (37). These features are difficult to recall accurately the following morning, which partly explains the growing interest in objective nocturnal monitoring. Together, these findings suggest that heat-sensitive itch in AD should be interpreted as a disorder of both peripheral input and central modulation. However, the extent to which altered central processing predicts nocturnal scratching, sleep disruption, or treatment response has not been adequately tested.

## 5 Clinical impact and patient-centered models of nocturnal scratching

### 5.1 Relationship between nocturnal pruritus and sleep architecture alterations

Nocturnal pruritus is not simply daytime itch occurring later. Video-based studies show that patients with severe AD may spend a substantial portion of the night scratching, with total scratching time exceeding 14% of recording time in some reports (38). Scratching is often more frequent in the first third of the night and occurs across all sleep stages, including REM and NREM sleep (39). In children, sleep continuity may be reduced, and the proportion of REM sleep altered, suggesting a fundamental disruption of restorative sleep processes (40). These observations give nocturnal scratching a different status: it is both a symptom and a behavior that can worsen the disease.

The physiological consequences of this sleep deficit include daytime somnolence, fatigue, and emotional distress, which are often overlooked in standard dermatological consultations (41). Studies have highlighted that sleep disturbance is significantly higher in AD patients than in healthy controls and worsens linearly with disease severity (42). The itch–scratch–sleep cycle creates a feedback loop where sleep deprivation lowers the threshold for pain and itch, further exacerbating the condition (43). Objective measurement of scratching during sleep is therefore clinically relevant because it may reveal a component of disease burden that is otherwise difficult to capture during routine assessment.

Although nocturnal scratching is closely associated with sleep disruption, establishing the causal direction is difficult: Itch may fragment sleep, but poor sleep may also lower sensory thresholds and amplify itch perception. Studies combining objective scratching, sleep-stage analysis, and patient-reported sleep quality are needed to clarify this bidirectional relationship.

### 5.2 Socioeconomic burden and quality of life impairment in adult and pediatric populations

The burden of AD extends beyond the individual patient, affecting families and the broader socioeconomic landscape. In a cross-sectional US sample, sleep disturbance was affected 66.9% of children aged 5–17 years with AD and, after adjustment for disease severity, itch intensity, and relevant sociodemographic factors, was associated with greater sleep-related impairment, depression, fatigue, anxiety, inattention, and impulsivity. These findings suggest that nocturnal itch and sleep disruption may adversely affect children's ability to engage in school-day activities, although their effects on objectively measured academic performance remain insufficiently characterized (44). More broadly, pediatric AD is associated with substantial impairment in dermatology-related quality of life and family quality of life, with greater disease severity generally associated with a higher burden (45, 46). Smartphone-based tracking systems have been proposed to help caregivers manage their children's symptoms more effectively by providing care reminders and symptom drawing tools (47). Among adults, the burden of AD may extend to work productivity and financial well-being. In a US National Health and Wellness Survey analysis, adults with AD had higher work absenteeism and activity impairment than propensity-matched non-AD controls, along with poorer health-related quality of life and higher self-reported rates of anxiety, depression, and sleep disorders (48). Similarly, a Swedish patient-reported survey identified both out-of-pocket costs and productivity losses, with greater itch intensity and activity impairment associated with worse health-related quality of life and higher costs (49). The impact of chronic itch on work productivity and daily activities imposes a significant economic cost, as highlighted in current clinical practice guidelines (50). Therefore, evaluating of the adult AD patient must include a thorough assessment of sleep loss and its impact on daily functioning (51). Guidelines emphasize the importance of asking general questions about itching and its persistence during every visit, as these factors are primary drivers of quality-of-life impairment. However, asking about these issues often sounds more precise than it is. Patients may normalize nocturnal scratching, forget episodes, or report only the worst nights. Objective measures can support treatment escalation when the visible skin examination underestimates the burden.

The socioeconomic burden of AD highlights the need for measurements that capture symptoms beyond visible skin inflammation. However, whether objective nocturnal scratch metrics improve treatment decisions, adherence, or caregiver burden compared with conventional assessments remains an important unanswered question.

### 5.3 Conceptual models for defining meaningful digital endpoints in clinical research

To bridge the gap between clinical observation and patient experience, researchers have developed patient-centered conceptual models for nocturnal scratching. Qualitative interviews and quantitative surveys have confirmed that itchy skin and scratching are the most burdensome aspects of AD, with approximately 65% of patients reporting nocturnal scratching at least once a week (52). This model identifies nocturnal scratching as a concept of interest that is directly related to meaningful aspects of health, such as sleep quality and emotional wellbeing. It has led to the development of evidence-based definitions for nocturnal scratching: a rhythmic and repetitive skin contact movement performed during a defined period of intended sleep (53). The establishment of these definitions is crucial for the standardization of digital health technologies. Emerging digital measures must be supported by evidence reflecting their relevance to patients, ensuring that the data collected—such as scratch frequency, duration, and intensity—correlate with the patient's lived experience (52). These conceptual models serve as a foundation for the next generation of clinical outcome assessments (COAs), moving the field toward more objective and patient-centric research.

A persistent difficulty in AD research is the gap between reported itch and observed scratching. Traditional patient-reported outcomes (PROs) often rely on 24-h recall, which can be inaccurate. However, new psychometric scales, such as the Scratch Intensity and Impact Scale and the Sleep-Related Itch and Scratch Scale, have demonstrated strong preliminary validity and reliability (54). Still, objective data remind us that some scratching occurs even in healthy individuals. The task is not to detect every movement but to interpret scratching in a clinical context.

Objective tools like the sheet-shaped body vibrometer (SBV) and wrist actigraphy (WA) have been compared to determine their relative strengths in capturing disease severity. While WA is often more suited for direct scratch evaluation, the SBV is preferable for assessing overall sleep efficiency and restlessness (55). Early methods for measuring itch, such as recording whole-body movement via vibration transducers on bed legs, established the basic assumption that nocturnal scratching is a reliable correlate of subjective itch (56). Modern studies continue to validate this, showing that increased severity of itch is accompanied by an increase in the number of scratching bouts rather than their individual duration (57). This nuanced understanding of scratching patterns is essential for refining the algorithms used in digital monitoring devices.

A digital scratch measure should be interpreted according to the stage of validation it has reached. First, sensor and algorithm performance concerns the technical ability to acquire and process relevant signals. Second, analytical validation determines whether the algorithm accurately identifies scratching against an appropriate ground truth, such as video annotation. Third, clinical validation examines whether the resulting metric is associated with clinically relevant aspects of AD, including itch severity, sleep impairment, daily functioning, and health-related quality of life. Fourth, longitudinal responsiveness evaluates whether the measure changes in a way that reflects treatment effects or clinically relevant changes in disease status. Finally, clinically meaningful thresholds, including meaningful within-patient change, are required before a digital metric can be interpreted as a patient-relevant endpoint. These stages may overlap during technology development, but evidence from one stage should not be interpreted as evidence from a subsequent stage.

## 6 Advances in digital technologies for objective scratching monitoring

Digital scratch-monitoring technologies should not be compared solely by the performance metrics reported in individual studies (Table 2). Sensitivity, specificity, precision, positive predictive value, and F1 score are influenced by the reference standard, scratch-event prevalence, study population, sample size, device placement, sampling frequency, recording environment, and the separation of training and test data. Accordingly, a high metric reported in one study does not necessarily indicate superior performance across populations or settings. The clinical value of a digital measure also depends on external validation, interpretability, responsiveness, and its association with patient-relevant outcomes.

**Table 2:** Selected digital technologies for objective nocturnal scratch monitoring in AD.

Technology	Main signal captured	Representative validation context and ground-truth method	Reported analytical performance and validation status	Key limitation	Potential role in AD care and research
Wrist actigraphy (activity proxy)	Wrist movement and acceleration	Adults and children with AD have worn actigraphs in home or clinical sleep studies; older studies generally assessed activity or sleep rather than video-defined scratching (8, 62)	Association with sleep disturbance or disease severity has been reported; direct scratch-classification accuracy was not established in early actigraphy studies	Non-scratch movement and finger-dominant scratching can confound wrist activity	Assessment of nocturnal activity and sleep disturbance; direct scratch measurement requires a validated scratch-specific algorithm
Smartwatch-based scratch detection	Accelerometer and gyroscope signals, sometimes linked to app-based reporting	Five adults with moderate AD were recorded during one inpatient night with simultaneous video annotation; subsequent home monitoring included 20 adults with AD (9)	For video-defined, non-weak scratches lasting at least 3 s, the initial study reported a PPV of 90.2% ± 6.6% and sensitivity of 84.6% ± 10.2%; no independent external-cohort validation was reported	The initial analytical validation was small and may miss weak, finger-only, or brief scratching	Patient-facing monitoring and remote symptom assessment are pending broader validation
Machine learning-enhanced actigraphy	Motion features processed by supervised or ensemble algorithms	Bilateral smartwatch accelerometer and gyroscope data from five patients with AD and two healthy volunteers were collected during two inpatient nights; infrared-video labels served as ground truth (58)	In subject-level hold-out testing, a feature-engineered gradient-boosting model reported a precision of 0.44, recall of 0.46, F1 score of 0.45, and accuracy of 0.84; no independent external-cohort validation was reported	Small inpatient development dataset, class imbalance, and subject-level variability limit generalizability	Development of analytically valid scratch-detection algorithms for clinical studies
Sensorized glove	Local hand or finger movement, acceleration, and contact patterns	A pilot study evaluated a glove combining finger stretch sensors and a palm inertial measurement unit in six children with AD; controlled testing was also performed (67)	The study reported 83% prediction accuracy in validation data, 99% in a separate 30-min controlled trial, and detection of 94.4% of scratching while the glove was worn; no independent external-cohort validation was reported	Glove wear, controlled testing, and the small pediatric pilot limit generalizability	Detailed characterization of scratching behavior and algorithm-validation studies
Skin-conformable wireless sensor	Local motion and skin-surface signals from a flexible dorsal-hand device	One device combined motion and acousto-mechanical signals in 11 predominantly pediatric patients with moderate-to-severe AD over 46 sleep nights; manually labeled infrared video was the reference standard (78)	Analytical accuracy was 99.0%, sensitivity 84.3%, specificity 99.3%, precision 82.5%, and F1 score 82.9%; no independent external-cohort validation was reported	Evidence is limited to one device and a small clinical cohort; adhesive placement and skin tolerance require consideration	Unobtrusive objective monitoring of nocturnal scratching, pending independent validation
Sheet-shaped body vibrometer	Bed or mattress vibration caused by body movement	Twenty outpatients with AD were measured at home for 5–10 nights and compared with wrist actigraphy; no simultaneous video or polysomnography was used (58)	The device quantified whole-body activity and sleep efficiency rather than directly observed scratching; no direct scratch-classification metric or independent external validation was reported	Activity includes scratching and other body movements, precluding direct inference about scratch duration	Assessment of nocturnal activity and sleep efficiency rather than direct scratch detection
Acoustic sensing	Scratch-related sound frequency and intensity	A wristwatch-type sound detector was compared with simultaneous video analysis in 24 measurements from healthy volunteers and people with AD (65)	Scratch time relative to sleep time was reported as almost identical between acoustic and video methods; sensitivity, specificity, PPV, and F1 score were not reported in the study abstract	Background noise and bedding or clothing materials may affect detection; no independent external-cohort validation was reported	Complementary signal for multimodal scratch-detection systems
Video recording with manual or automated annotation	Visual confirmation of scratching behavior	Direct observation during intended sleep; 35 adults with AD were recorded over 112 nights in a representative study (38)	Common reference standard for analytical validation; scratch time varied with AD severity in the representative study, but video is not a scalable long-term endpoint	Privacy concerns, annotation burden, and limited feasibility for prolonged home monitoring	Algorithm training, benchmarking, and validation of emerging technologies

AD, atopic dermatitis; F1 score, harmonic mean of precision and recall. Performance metrics are reported only when available in the source studies and should not be directly compared across studies because of differences in sample characteristics, scratch-event prevalence, reference-standard procedures, algorithms, sensor placement, and recording environments. Analytical validation does not establish clinical validity, responsiveness, or clinically meaningful thresholds.

### 6.1 Wearable sensors and wrist-worn actigraphy with machine learning algorithms

Wrist-worn actigraphy has become the most common method for objective scratching monitoring due to its convenience and the ubiquity of smartwatches. Recent advances have focused on developing machine learning algorithms that can distinguish scratching from other nocturnal movements like turning over or adjusting blankets. In an initial smartwatch study, the Itch Tracker app reported a positive predictive value exceeding 90% against video monitoring (9). However, this result should be interpreted in the context of the study population, scratch-event prevalence, reference-standard procedure, and validation setting; it should not be generalized as a device-independent estimate of performance. Similarly, researchers have used digital wrist actigraphy to quantify nocturnal scratch duration and intensity, achieving F1 scores that suggest the potential for real-world application (58).

The development of these digital measures often involves a hierarchical paradigm: first determining the intended sleep window, then detecting sleep-wake states, and finally identifying scratching episodes (59). Validation studies in adults with AD have shown that hand-mounted sensors can accurately capture scratching movements, although challenges remain regarding the generalizability of these models to diverse populations (60). Some approaches use topological features and model-ensembling to improve accuracy, addressing the problem of imbalanced data where scratching events are relatively rare compared with other movements (61). Despite these advances, early methods like simple wrist activity monitors still provide a baseline for comparison, reminding researchers that selecting scratching activity exclusively remains a complex signal-processing task (62).

Although wrist-worn devices are among the most scalable approaches for home monitoring, their performance depends heavily on algorithm design, training data quality, and the ability to distinguish scratching from other nocturnal movements. Cross-population validation remains essential before these tools can be accepted as reliable clinical or trial endpoints.

### 6.2 Non-wearable solutions including thermal imaging and sheet-shaped body vibrometers

While wearables are popular, they can be uncomfortable for patients with sensitive skin or may be removed during sleep. Non-wearable, touchless solutions offer a promising alternative. Thermal camera technology has been used to quantify scratching by detecting the heat generated by friction and the exposure of warmer underlying skin layers. This system allows for the annotation of dominant versus non-dominant hand scratching, revealing that AD patients often scratch more with their dominant hand (63). Another innovative approach involves the use of SBV placed under the mattress. It detects the specific vibrations associated with scratching, and new algorithms have been developed to avoid overestimating scratching time by excluding general body movements (64). Non-wearable approaches may reduce patient burden, particularly for those who cannot tolerate sensors on inflamed skin. However, their scalability, privacy acceptability, and robustness across different home environments require further validation before they can be widely used as clinical or trial endpoints.

### 6.3 Multimodal sensing approaches integrating acoustic analysis and bio-electrode data

To increase the precision of scratch detection, researchers are increasingly turning to multimodal sensing. This involves combining different types of data, such as motion (IMU) and sound (acoustic). Acoustic evaluation systems use wristwatch-type sound detectors to record the specific frequency and energy of scratching sounds, which are then processed by software to evaluate the movement (65). This method is significantly faster than manual video analysis and has shown high correlation with visual ground truth.

Innovative form factors, such as multimodal sensing rings and sensorized gloves, have also been developed. A sensing ring consisting of an accelerometer and a contact microphone can estimate scratch intensity on a milliwatt power scale, providing a more granular view of the itch–scratch cycle than simple duration (66). Sensorized gloves, like the SIGMA system, use microtubular stretchable sensors on each finger to detect fine-motor scratching that might be missed by a wristband (67). These technologies have even been adapted for animal models, where automated acoustic detection of mouse scratching helps accelerate the development of new anti-itch therapies (68).

Multimodal sensing may improve detection accuracy by combining complementary signals, but greater technical complexity does not automatically translate into clinical usefulness. Future work should determine whether multimodal systems provide information beyond scratch duration that meaningfully improves phenotyping, treatment monitoring, or patient-centered outcomes.

### 6.4 Analytical validation and standardization of digital health technologies in home settings

An important implementation challenge for digital scratching monitoring is analytical validation in real-world home settings; this is necessary but not sufficient for clinical validation. Studies comparing wearable wristbands (like the GENEActiv) with touchless sensors (like the Emerald radio frequency sensor) have shown that both can accurately quantify the sleep window and scratching events in a patient's own bedroom (7). However, the performance of these devices can vary, and there is a recognized need for larger studies to validate data analysis algorithms across target populations (69). The variety of new methods—from thermal imaging to acoustic analysis—is welcomed, but it necessitates a focus on standardization to ensure that data can be used for clinical decision-making (56, 70).

Digital technologies may contribute to a gradual shift toward monitoring-feedback models in AD care, although their integration into routine diagnosis and treatment requires further validation (71). This loop is enhanced by artificial intelligence-assisted analysis and telemedicine platforms that integrate real-time data. Electromagnetic sensing techniques, including radio frequency reflectometry, offer future possibilities for non-invasive monitoring of biomarkers like trans-epidermal water loss (TEWL) alongside scratching (72). Electrical impedance spectroscopy provides an impedance-based assessment of skin barrier properties. In children with AD, electrical impedance spectroscopy has been reported to detect barrier dysfunction and reflect changes in barrier integrity during dupilumab treatment (73, 74). Tape stripping provides a minimally invasive approach to sampling the stratum corneum and assessing epidermal barrier-related and inflammatory biomarkers. In AD, tape-strip samples may be used to quantify proteins, lipids, cytokines, chemokines, and other molecular signatures associated with keratinocyte activation and cutaneous inflammation (75). Although tape stripping does not directly measure scratching or thermally provoked itch, combining tape-strip biomarkers with TEWL, electrical impedance spectroscopy, environmental exposure, patient-reported itch, sleep measures, and digital scratch monitoring may help define the biological context of nocturnal scratching in future longitudinal studies. Furthermore, the use of these tools to monitor the effects of medications, such as the non-sedating antihistamine olopatadine, demonstrates their practical value in assessing treatment efficacy without affecting sleep quality (76). Skin-conformable wireless sensors represent an advanced but still developing approach to objective symptom quantification, with potential value for continuous monitoring if their performance and usability are validated in real-world AD populations (77).

At present, the main barrier is no longer whether scratching can be detected, but whether digital scratch metrics are reproducible, interpretable, and clinically meaningful across real-world settings. Transparent algorithms, standardized definitions, and validation across age groups, skin severities, and sleep environments will be necessary before these measures can be widely adopted.

### 6.5 Confounding and design considerations for studies of thermal exposure and nocturnal scratching

The relationship between thermal exposure and nocturnal scratching cannot be interpreted as a temperature-specific effect without careful consideration of coexposures, confounding, and effect modification. Environmental conditions, including temperature, humidity, sweating, and nocturnal occlusion, have been associated with symptom variation in AD and nocturnal itch; however, their independent contributions remain difficult to disentangle (78, 79). Baseline AD severity, lesion location, age, medication use, topical treatment, season, and pre-existing skin-barrier status may confound associations between thermal conditions and scratching. Bedroom humidity, bedding, clothing, air-conditioning use, and physical activity before sleep may influence both thermal exposure and nocturnal symptoms and should be measured separately where feasible. Not all relevant variables should be treated as confounders in every analysis. Depending on the temporal sequence and causal question, sweating, skin temperature, sleep stage, and barrier disruption may function as mediators or effect modifiers rather than baseline confounders (80). Adjusting for variables that lie on the causal pathway may obscure clinically relevant mechanisms. Future studies should therefore prespecify their causal question and use an explicit conceptual model to distinguish confounders, coexposures, mediators, and effect modifiers (40, 81). Prospective within-person studies should repeatedly measure ambient temperature and humidity, skin temperature, bedding and clothing conditions, scratching, sleep, patient-reported itch, disease severity, treatment exposure, and barrier-related measures. Home-based studies combining wearable and touchless technologies illustrate the feasibility of collecting several of these domains simultaneously, although dedicated studies of thermal exposure remain needed (70). Such designs may help determine whether thermal exposure is independently associated with objectively measured nocturnal scratching and whether this association differs according to disease activity, age, barrier status, or treatment.

### 6.6 Research gaps and a prospective validation framework

The central unanswered question is whether quantified thermal exposure is independently associated with objectively measured nocturnal scratching in AD. Addressing this question requires prospective, repeated within-person measurements rather than cross-sectional comparisons of patient-reported triggers. A future home-based study could recruit children and adults across a range of AD severities and repeatedly collect bedroom temperature and relative humidity, skin temperature, bedding and clothing conditions, wearable or contactless scratch data, and objective sleep measures. These data should be linked with daily patient-reported itch and sleep outcomes, clinical AD severity assessments, treatment and topical therapy exposure, and complementary measures of barrier status, such as TEWL or electrical impedance spectroscopy (52, 73, 74). Tape-strip biomarkers could be incorporated into a nested mechanistic component where feasible (75). The primary analysis should examine whether time-varying thermal exposure predicts subsequent nocturnal scratching within individuals after accounting for prespecified confounders and coexposures. Secondary analyses should assess whether the association differs by age, disease severity, lesion distribution, barrier status, or treatment, and whether changes in scratch metrics correspond to patient-reported itch, sleep impairment, and clinically meaningful improvement. This design would not presume that thermal exposure causes scratching; rather, it would provide a framework for testing the proposed association, identifying potential effect modifiers, and establishing whether digital scratch measures have patient-relevant clinical value.

## 7 Conclusion and future perspectives

Thermally provoked itch in AD arises from the interaction of environmental triggers, barrier dysfunction, neuroimmune activation, and altered sensory processing. TRPV1/TRPV3 signaling, epithelial mediators, fibroblast-derived artemin, neuropeptides, and altered peripheral and central sensory processing provide a plausible mechanistic framework for thermally provoked itch. However, these pathways should not be interpreted as an established unified mechanism in human AD, because direct evidence varies substantially across individual components.

Digital nocturnal scratch monitoring offers an objective behavioral measure that may complement patient-reported assessments of itch and sleep. Wearable, contactless, acoustic, and multimodal devices have shown that nocturnal scratching can be detected under specific validation conditions. However, analytical performance should not be conflated with clinical validity, responsiveness to treatment, or relevance to thermal exposure. Direct evidence that quantified thermal exposure changes objectively monitored nocturnal scratching in patients with AD remains limited. Digital scratch-monitoring tools should transition from technical performance and analytical validation to clinical validation, longitudinal responsiveness, and the establishment of clinically meaningful change thresholds. Currently, most available evidence pertains to the first two stages.

Future studies should use prospective within-person designs that integrate continuous ambient temperature and humidity monitoring, skin temperature, objective scratch and sleep measurements, patient-reported itch and sleep outcomes, disease severity, and treatment information. Such studies should consider key confounders, including baseline disease severity, lesion location, medication use, bedding and clothing, sweating, sleep stage, season, and air-conditioning use. This approach may determine whether thermal exposure independently predicts clinically meaningful nocturnal scratching and whether a reproducible heat-responsive clinical characteristic can be identified.
